# Tropical Atlantic temperature seasonality at the end of the last interglacial

**DOI:** 10.1038/ncomms7159

**Published:** 2015-01-22

**Authors:** Thomas Felis, Cyril Giry, Denis Scholz, Gerrit Lohmann, Madlene Pfeiffer, Jürgen Pätzold, Martin Kölling, Sander R. Scheffers

**Affiliations:** 1 MARUM—Center for Marine Environmental Sciences, University of Bremen, 28359 Bremen, Germany; 2 Institute for Geosciences, Johannes Gutenberg University Mainz, 55099 Mainz, Germany; 3 Alfred Wegener Institute, Helmholtz Centre for Polar and Marine Research (AWI), 27570 Bremerhaven, Germany; 4 Marine Ecology Research Centre, Southern Cross University, Lismore, New South Wales 2480, Australia

## Abstract

The end of the last interglacial period, ~118 kyr ago, was characterized by substantial ocean circulation and climate perturbations resulting from instabilities of polar ice sheets. These perturbations are crucial for a better understanding of future climate change. The seasonal temperature changes of the tropical ocean, however, which play an important role in seasonal climate extremes such as hurricanes, floods and droughts at the present day, are not well known for this period that led into the last glacial. Here we present a monthly resolved snapshot of reconstructed sea surface temperature in the tropical North Atlantic Ocean for 117.7±0.8 kyr ago, using coral Sr/Ca and δ^18^O records. We find that temperature seasonality was similar to today, which is consistent with the orbital insolation forcing. Our coral and climate model results suggest that temperature seasonality of the tropical surface ocean is controlled mainly by orbital insolation changes during interglacials.

## Abstract (web summary)

The last interglacial has been suggested as a test bed for models developed for future climate prediction, yet many climatic parameters remain unknown. Here, the authors present a precisely dated fossil coral and show that temperature seasonality in the southern Caribbean Sea 118 ka was similar to today.

## Graphical abstract

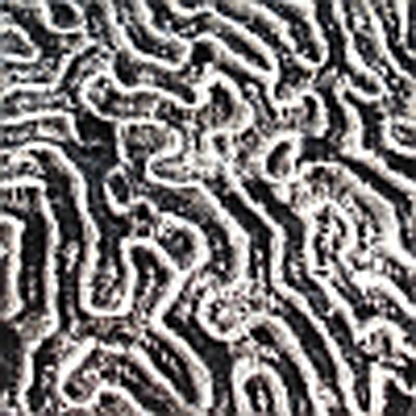

The last interglacial, although not a direct analogue for future climate, has received much attention in the climate-modelling community^1^,^2^,^3^ and has been suggested as a test bed for models developed for future climate prediction^2^,^4^. This period (~127–117 kyr ago) was characterized by strong orbital insolation forcing^5^, relative warmth^6^ and high sea level^7^. In the Northern Hemisphere, changes in the Earth’s orbit around the sun led to a stronger seasonality of insolation compared to today^5^, which resulted in increased temperature seasonality at the Earth’s surface as inferred from proxy records^8^,^9^,^10^ that commonly represent the time interval of maximum seasonal insolation forcing^5^ between ~127 and ~124 kyr ago. In contrast, the temperature seasonality at the end of the last interglacial (~118 kyr ago), when Northern Hemisphere insolation seasonality was close to today’s value^5^, is not well known. This period that led into the last glacial is particularly interesting as it was characterized by catastrophic collapse of polar ice sheets and substantial sea-level rise^11^,^12^, abrupt changes in ocean circulation^13^,^14^ and large-scale climate perturbations^15^. It has been suggested that the end of the last interglacial may provide clues to a better understanding of the potential for rapid ice-sheet collapse and sea-level rise and, consequently, for abrupt perturbations of the ocean–atmosphere system, under future climate change^11^,^12^,^14^. At the present day, the seasonal temperature changes of the tropical ocean play an important role in seasonal climate extremes such as hurricanes, floods and droughts^16^,^17^,^18^,^19^. A better understanding of the temperature seasonality ~118 kyr ago is, thus, essential to establish a baseline to evaluate the seasonal response in climate model simulations, for both the end of the last interglacial and for projections of future climate change.

Here we investigate the monthly resolved Sr/Ca and δ^18^O environmental proxy signals in a precisely dated shallow-water fossil coral recovered from the southern Caribbean and reconstruct the temperature seasonality in the surface waters of the tropical North Atlantic Ocean at the end of the last interglacial. Sr/Ca variations in aragonitic coral skeletons are a proxy for temperature variability^20^, which has previously been successfully applied to last interglacial fossil corals^9^,^10^,^21^. Coral δ^18^O, a proxy that reflects both temperature and seawater δ^18^O variations, is used to support our reconstruction. The ^230^Th/U method allows precise dating of corals that grew during the last interglacial period^22^. Our findings indicate that temperature seasonality in the southern Caribbean Sea at 118 kyr ago was similar to today. Our coral records and simulations with a coupled atmosphere–ocean general circulation model indicate an orbital control on temperature seasonality in the tropical North Atlantic at the end of the last interglacial, despite the large-scale perturbations of ocean circulation and climate during this period, and suggest that temperature seasonality of the tropical surface ocean is controlled mainly by orbital insolation changes during interglacials.

## Results

### Coral preservation and age

**Figure 1:**
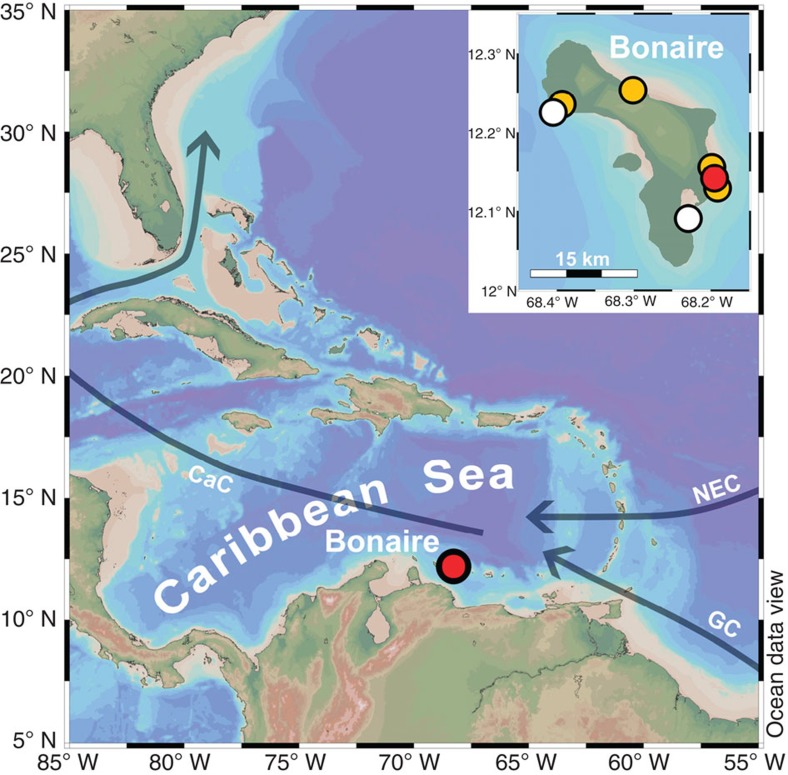
Map of the western tropical North Atlantic Ocean. The location of our coral site at Bonaire in the southern Caribbean Sea and surface ocean circulation patterns in the study area (Guyana Current, GC; Caribbean Current, CaC; North Equatorial Current, NEC) are indicated. Bonaire is situated off the continental shelf of South America in open-ocean waters. The inset shows the locations of our last interglacial (red circle, this study), Holocene^23^,^27^ (orange circles) and modern^23^,^27^ (white circles) coral sites at Bonaire.

The fossil shallow-water coral (*Diploria strigosa*) was recovered at Bonaire, an open-ocean island in the southern Caribbean Sea, located ~100 km north of South America and ~300 km northwest of the Cariaco Basin (Fig. 1). Bonaire is situated off the South American continental shelf in the northwestward-flowing Caribbean Current, an extension of the Guyana Current that transports equatorial Atlantic surface waters along northeastern South America towards the Caribbean Sea. Thus, sea surface temperature (SST) at Bonaire is representative for a large area of the tropical North Atlantic Ocean^23^. Bonaire is influenced by the easterly trade winds, and its present-day climate is semi-arid with an annual precipitation of ~550 mm and the main rainy season during boreal winter^24^. Bonaire is not influenced by the seasonally migrating Intertropical Convergence Zone (ITCZ) because the northernmost ITCZ position that is reached during boreal summer is located south of Bonaire, over northern South America and the Cariaco Basin^25^. The fossil coral colony (BON-5-D) was drilled in growth position on top of an elevated reef terrace at the eastern coast of Bonaire (Washikemba). The coral site (68° 11.765′ W, 12° 8.246′ N) is at ~1.5 to ~2.0 m above present sea level, in a distance of ~50 m from the present-day sea cliff. Nearby *D. strigosa* colonies in growth position (<6 m distance) suggest that this fossil coral community is preserved *in situ*. X-radiography, powder X-ray diffraction, thin-section petrography and scanning electron microscope analysis indicate that the fossil coral is very well preserved (Methods and Supplementary Figs 1–3). ^230^Th/U dating yielded a coral age of 117.7±0.8 kyr, showing that the colony grew at the end of the last interglacial period. The initial (^234^U/^238^U) activity ratio is in agreement with the (^234^U/^238^U) of modern seawater, providing strong confidence for the reliability of the coral age (Methods and Supplementary Table 1).

### Coral-based SST seasonality reconstruction

**Figure 2:**
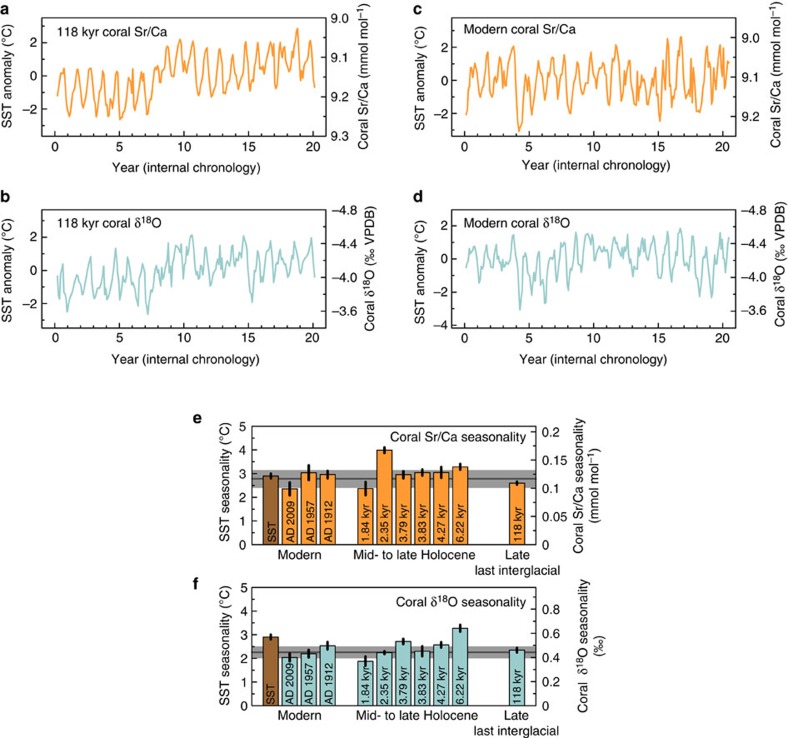
Tropical North Atlantic coral-based temperature seasonality. (**a**) Monthly Sr/Ca record of a fossil Bonaire *Diploria strigosa* coral that grew at 117.7±0.8 kyr ago for 20 years in southern Caribbean Sea surface waters. (**b**) The monthly coral δ^18^O record. (**c**) Monthly Sr/Ca record of a modern Bonaire *D. strigosa* coral that grew around AD 1912. (**d**) The monthly coral δ^18^O record. (**e**) Sr/Ca-based sea surface temperature (SST) seasonality from Bonaire *D. strigosa* corals for snapshots since 118 kyr ago, based on monthly records comprising a total of 315 years, and Bonaire instrumental SST seasonality (1910–2000, 2° × 2° gridbox centred at 12° N, 68° W, ERSST.v3b)^26^. The dark grey line represents the reconstructed modern mean SST seasonality based on three modern corals and the light grey bar the ±1 s.d. around this mean. (**f**) The coral δ^18^O-based SST seasonality. Deviations from Sr/Ca- and instrument-based estimates are due to seasonal seawater δ^18^O effects. Coral-based SST anomalies (corresponding mean value was subtracted) (**a**–**d**) and SST seasonalities (**e**,**f**) are derived from seasonal Sr/Ca-SST (−0.042 mmol mol^−1^ per °C) and δ^18^O-SST relationships (−0.196‰ per °C) for *D. strigosa*^37^. The uncertainty assigned to each SST seasonality estimate is the ±1 s.e. Holocene and modern coral data are from refs 23, 27.

The 118-kyr-old Bonaire coral provides a monthly resolved snapshot of tropical Atlantic SST variability for a time window of 20 years at the end of the last interglacial. This is substantially longer than the only other seasonally resolved snapshot of tropical Atlantic SST for the last interglacial, an ~5-year record of a 127-kyr-old coral from Isla de Mona (67.9° W, 18.1° N) in the northern Caribbean Sea^9^. Our Bonaire monthly resolved coral Sr/Ca- and δ^18^O-SST reconstructions show clear annual cycles in both proxies (Fig. 2a,b), giving additional confidence that the analysed coral skeleton was not subject to diagenetic alteration. The Sr/Ca-SST reconstruction indicates a seasonality of 2.6±0.1 °C (±1 s.e.) at 118 kyr ago (Fig. 2a,e). Monthly resolved records of three modern Bonaire *D. strigosa* corals satisfactorily document the instrumental SST^26^ seasonality of 2.9±0.1 °C (±1 s.e.; 1910–2000), indicating a reconstructed modern Sr/Ca-SST seasonality that ranges from 2.4±0.3 °C (±1 s.e.) to 3.0±0.3 °C (±1 s.e.) for time intervals of the last century, resulting in a reconstructed modern mean seasonality of 2.8±0.4 °C (±1 s.d.; ref. 23; Fig. 2c,e). Taking into account these differences in the reconstructed SST seasonality among the three modern corals indicates that the reconstructed SST seasonality of 2.6±0.1 °C (±1 s.e.) at 118 kyr ago, at the end of the last interglacial, is not significantly different from today (Methods and Supplementary Note 1).

The coral δ^18^O-SST reconstruction for 118 kyr ago indicates a seasonality of 2.4±0.1 °C (±1 s.e.), which is very similar to the Sr/Ca-based seasonality estimate of 2.6±0.1 °C (±1 s.e.; Fig. 2a,b,e,f). Thus, the coral δ^18^O seasonality at 118 kyr ago may be attributed mainly to the seasonality of SST. This is broadly in line with the modern situation^27^, where the mean SST seasonality reconstructed by coral δ^18^O of 2.3±0.3 °C (±1 s.d.) is slightly reduced (by ~0.5 °C, not correcting for seasonal seawater δ^18^O changes) relative to the Sr/Ca- and instrument-based estimates (Fig. 2e,f), most likely owing to hydrologic cycle effects such as the Bonaire winter rainfall regime^24^. The coral δ^18^O-SST reconstruction supports our major finding based on coral Sr/Ca, and both proxies indicate SST seasonality in the southern Caribbean Sea at the end of the last interglacial similar to today. Consequently, both proxies may also suggest a Bonaire hydrologic cycle similar to today at 118 kyr ago. Crucially, our results are robust towards the choice of the coral Sr/Ca-SST and δ^18^O-SST relationships, which affect mainly the absolute magnitude of reconstructed SST seasonality but have only minor effect on the relative seasonality estimates among corals, and we would have reached identical conclusions using other relationships (Supplementary Fig. 4).

## Discussion

**Figure 3:**
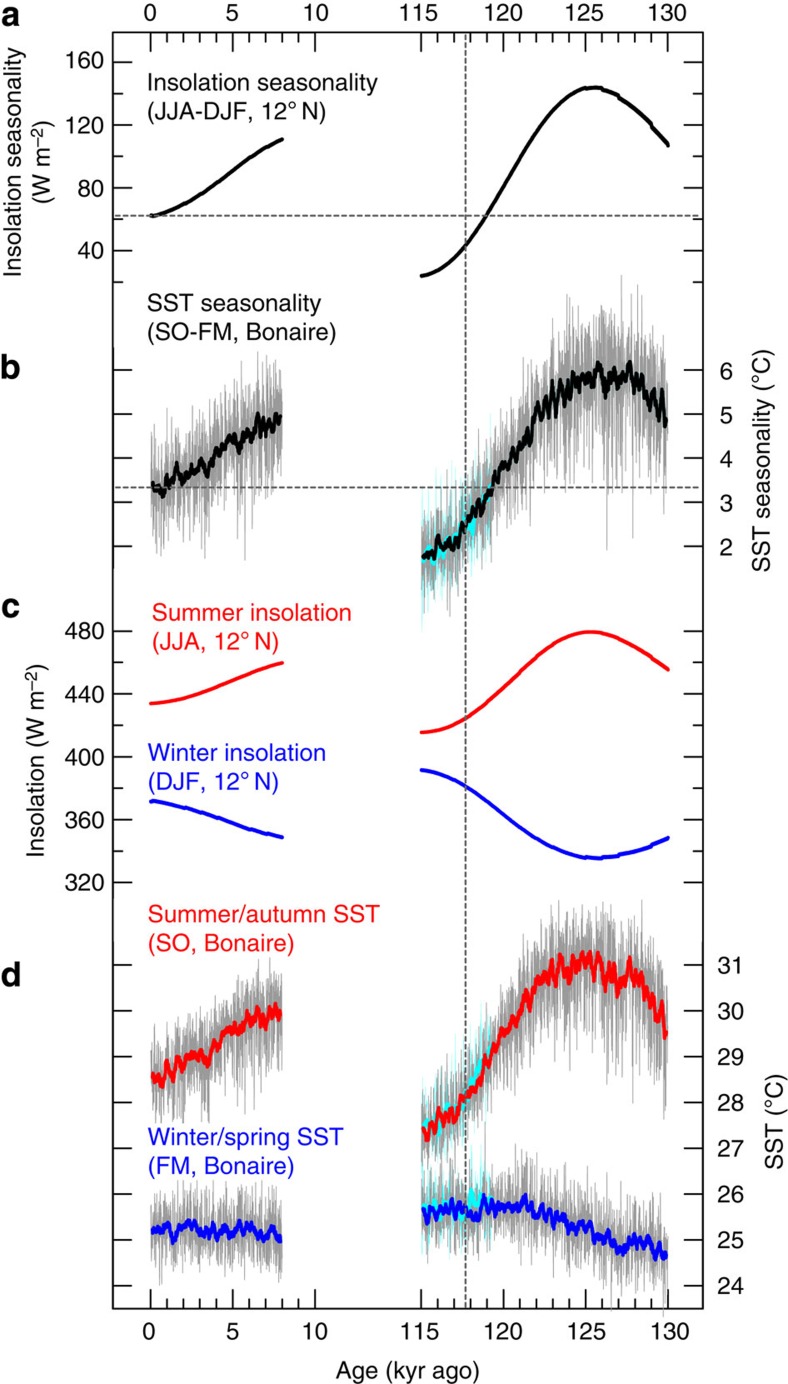
Tropical North Atlantic insolation and temperature changes. (**a**) Insolation seasonality^5^ at the latitude of Bonaire, calculated as difference of boreal summer (June–July–August, JJA) minus winter insolation (December–January–February, DJF). (**b**) Sea surface temperature (SST) seasonality at Bonaire simulated by the coupled atmosphere-ocean general circulation model COSMOS (1° × 1° gridbox centred at 12.5° N, 68° W), derived from the difference of simulated summer/autumn (September–October, SO) minus winter/spring (February–March, FM) SST. The SST seasonality evolution is very similar to that derived from the difference of warmest minus coolest SST (Supplementary Fig. 7). (**c**) Summer (JJA) and winter (DJF) insolation^5^ at the latitude of Bonaire. (**d**) Summer/autumn (SO) and winter/spring (FM) SST at Bonaire simulated by COSMOS. βold line (**b**,**d**) represents a 21-point running average, representing an average of 210 calendar years. Results of the freshwater hosing experiment are also shown (light blue). Dashed horizontal lines (**a**,**b**) indicate the modern value for insolation and simulated SST seasonality. Dashed vertical line indicates the Bonaire coral age (117.7±0.8 kyr).

The annual SST cycle in the Caribbean Sea, with a minimum in boreal winter/spring and a maximum in boreal summer/fall, follows primarily the annual cycle of insolation^28^,^29^. Bonaire monthly coral Sr/Ca records for snapshots since the mid-Holocene, comprising a total length of 295 years, suggest that the SST annual cycle in the southern Caribbean Sea has not substantially changed, with the exception of a time interval at 2.35 kyr ago^23^ (Fig. 2e). Disregarding the 2.35 kyr coral, a trend towards lower SST seasonality during the time interval 6.22–1.84 kyr ago may be inferred from the coral Sr/Ca records, as well as a slightly but significantly higher SST seasonality than the present day at 6.22 kyr ago. Such an evolution through time would be consistent with an orbital insolation control on Holocene SST seasonality in the southern Caribbean Sea (Fig. 3a), which is supported by simulations with a coupled atmosphere–ocean general circulation model (Community Earth System Models, COSMOS; Methods and Fig. 3b). However, we note that the magnitude of the trend in the fossil coral data is minor, close to the ±1 s.d. range of the modern mean Sr/Ca-SST seasonality reconstructed from three modern corals (Fig. 2e), which may also reflect the relatively small magnitude of insolation-controlled SST seasonality changes at lower latitudes throughout the Holocene (Fig. 3a,b).

Similarly, the coral δ^18^O-SST reconstruction^27^ reveals a trend towards lower seasonality during the time interval 6.22–1.84 kyr ago, which is more pronounced compared with the trend that may be inferred from coral Sr/Ca, as well as a substantially and significantly higher seasonality than the present day at 6.22 kyr ago (Fig. 2f). This evolution of coral δ^18^O seasonality through time is consistent with an insolation control on Holocene SST seasonality in the southern Caribbean Sea (Fig. 3a,b). Differences between the coral δ^18^O-SST and Sr/Ca-SST seasonality estimates (Fig. 2e,f) reflect primarily seasonal changes in seawater δ^18^O; however, we note that reconstructions of seawater δ^18^O seasonality can be sensitive towards the choice of the coral δ^18^O-SST and Sr/Ca-SST relationships (Supplementary Fig. 4). However, for 6.22 kyr ago, an anomalous seawater δ^18^O seasonality may be inferred from the coral records that could be explained by hydrologic cycle effects such as, among others, Bonaire summer rainfall^27^, which would be contrary to the present-day winter rainfall regime^24^ (Supplementary Fig. 4). This interpretation would be in line with reconstructions of increased summer rainfall over northernmost South America during the early to mid-Holocene, owing to a more northerly position of the boreal-summer ITCZ^30^ and possibly paired with a thermodynamic increase in rainfall because of strengthening local summer insolation^31^. We note that the subsequent southward migration of the boreal-summer ITCZ over the course of the Holocene that was controlled by orbital insolation changes^30^,^31^ is also in line with the trend towards lower coral δ^18^O seasonality over this time interval (Fig. 2f).

The significantly increased SST seasonality at 2.35 kyr ago, indicated by coral Sr/Ca (Fig. 2e), may be related to internal climate variability and is interpreted to reflect a time interval of strengthened El Niño-Southern Oscillation (ENSO) teleconnections to the Caribbean region^23^, probably modulated by the North Atlantic Oscillation (NAO). This interpretation is broadly in line with the present-day modulation of southern Caribbean SST seasonality by ENSO teleconnections^23^,^32^, which vary in strength on interdecadal timescales and are modulated by the NAO^33^. Indeed, pronounced interannual variability at a period of 5.7 years in the Sr/Ca record of the 2.35 kyr coral^23^, the most prominent period in the cospectrum of the instrumental indices of ENSO and NAO^34^,^35^, may be indicative of pronounced ENSO–NAO interactions at that time^23^. Importantly, the strength of the ENSO phenomenon in the tropical Pacific did not change markedly around 2.3 kyr ago^36^. We note that the increased coral Sr/Ca-SST seasonality at 2.35 kyr ago is not accompanied by an increased coral δ^18^O-SST seasonality (Fig. 2e,f), which suggests an anomalous seawater δ^18^O seasonality that could be explained by hydrologic cycle effects such as, among others, increased Bonaire winter rainfall (Supplementary Fig. 4). This interpretation would be broadly in line with the present-day modulation of Bonaire climate by ENSO teleconnections, where La Niña events result in increased SST seasonality through anomalous winter cooling^23^ as well as in increased winter rainfall^24^.

Our coral-based finding of SST seasonality similar to today in the southern Caribbean Sea at 118 kyr ago (Fig. 2e) is consistent with an insolation seasonality at the latitude of Bonaire that was close to today’s value (Fig. 3a). This result could be interpreted in a way that southern Caribbean SST seasonality at that time was controlled mainly by orbital insolation changes. Simulations performed with a coupled atmosphere–ocean general circulation model (COSMOS) support this interpretation (Methods). The modelled changes in southern Caribbean SST seasonality at Bonaire throughout the last interglacial follow largely the variations in insolation forcing over the time interval 130–115 kyr ago (Fig. 3). Moreover, the modelled global surface air temperature anomaly indicates that temperature seasonality in the southern Caribbean at 118 kyr ago is part of a hemisphere-scale pattern that can be attributed largely to insolation forcing (Supplementary Fig. 5). Additional model simulations with freshwater forcing to mimic an abrupt ice-sheet collapse and weakening of the North Atlantic thermohaline circulation at 118 kyr ago or with reduced Greenland ice sheet and dynamic vegetation reveal very similar results (Methods), indicating no significant impact on southern Caribbean SST seasonality (Fig. 3 and Supplementary Figs 5 and 6). Thus, our model-based results strongly suggest that SST seasonality in the tropical North Atlantic Ocean at the end of the last interglacial was controlled mainly by orbital insolation changes. Although the slightly lower modelled SST seasonality at 118 kyr ago relative to today (Fig. 3b) appears to be consistent with the coral Sr/Ca-SST seasonality estimate for the end of the last interglacial (Fig. 2e), we consider the latter as similar to today as a result of our uncertainty assignments that take into account the differences in the seasonality estimates among the three modern corals (Methods).

**Figure 4:**
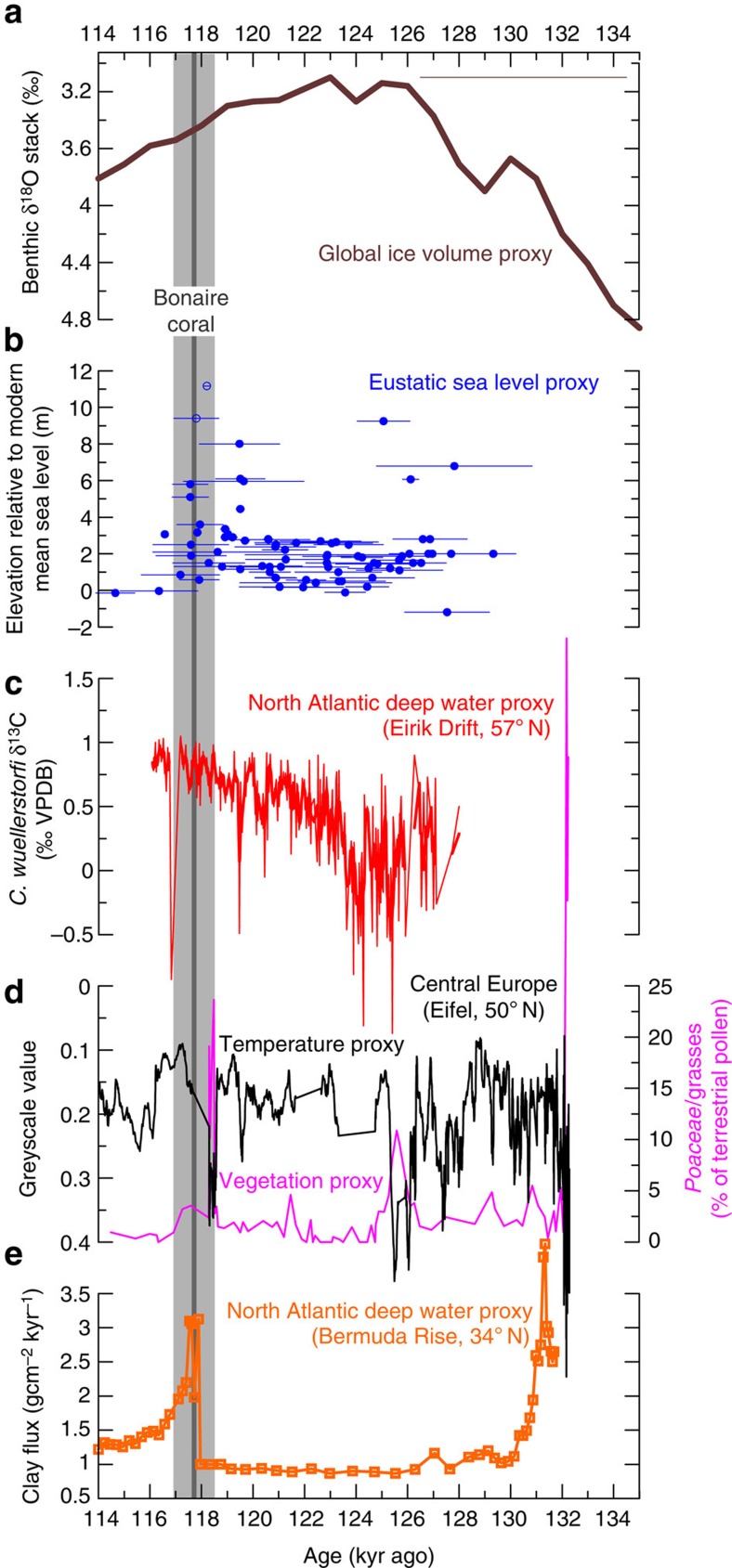
Bonaire coral age and last interglacial sea level and climate change. (**a**) LR04 stack of globally distributed benthic δ^18^O records, reflecting global ice volume changes^66^. (**b**) Relative sea level from Western Australian corals, indicating eustatic sea level rose to ~8 m above present at 118.1±1.4 kyr ago^12^. Open symbols indicate corals collected not *in situ* or affected by tectonic uplift^12^. (**c**) North Atlantic epibenthic foraminiferal δ^13^C record, indicating pronounced reductions in North Atlantic Deep Water production (bottom water δ^13^C reductions) at ~119.5 and ~116.8 kyr ago^14^. Bold line indicates a 3-point running average. (**d**) Eifel Laminated Sediment Archive greyscale stack from maar lakes in Germany, indicating a prominent cold and arid event at 118 kyr ago that was accompanied by high grass pollen abundance^15^. (**e**) Clay flux record from excess ^230^Th-measurements in North Atlantic sediments indicating a rapid increase in recirculation-derived clay supply (and the proportion of southern source water) at ~118 kyr ago, associated with a cessation in North Atlantic deep water flow^13^. The dark grey line indicates the Bonaire coral age (117.7 kyr) and the light grey shading the corresponding 2σ uncertainty (±0.8 kyr). Both Bonaire coral and sea-level jump^12^ were dated by the ^230^Th/U-method, whereas the sediment records^13^,^14^,^15^,^66^ were not absolutely dated. Age uncertainty is shown as reported in original publication, if available.

The relatively stable SST seasonality in the tropical North Atlantic Ocean at the end of the last interglacial and its inferred orbital control is remarkable as this period was characterized by large-scale perturbations of ocean circulation and climate resulting from instabilities of polar ice sheets^11^,^12^,^13^,^14^,^15^. Results from Western Australia suggest that, after a prolonged period of stable sea level at ~3–4 m above present sea level between 127 and 119 kyr ago, eustatic sea level rose rapidly to ~8 m above present at the end of the last interglacial, peaking at 118.1±1.4 kyr ago^12^, which is contemporaneous with the age of our southern Caribbean coral (117.7±0.8 kyr; Fig. 4b). It has been suggested that this substantial jump in sea level at the end of the last interglacial resulted from collapse of the Greenland and particularly Antarctic ice sheets, after a critical ice-sheet stability threshold was crossed^12^. Such an event may have had substantial impacts on global ocean circulation and climate. Interestingly, varved lake sediments in central Europe indicate an extreme 468-year arid and cold event at 118 kyr ago (Fig. 4d), which has been interpreted to result from a sudden southward shift of the warm North Atlantic drift^15^. Furthermore, western North Atlantic sediments indicate an abrupt ~400-year deep-water reorganization event at ~118 kyr ago associated with changes in the thermohaline circulation^13^ (Fig. 4e), which has been interpreted to mark the beginning of climate deterioration at the end of the last interglacial^13^. Recent evidence suggests even two events of substantial North Atlantic deep-water reduction at the end of the last interglacial, at ~119.5 and ~116.8 kyr ago^14^ (Fig. 4c).

Our findings based on combining coral proxy records with climate model simulations indicate that northern tropical Atlantic SST seasonality at 118 kyr ago was similar to today and controlled mainly by orbital insolation changes, despite dramatic ocean circulation and climate perturbations resulting from instabilities of polar ice sheets that characterized the end of the last interglacial^11^,^12^,^13^,^14^,^15^. Today, tropical Atlantic SST plays a major role in seasonal climate extremes, such as hurricanes, flashfloods and droughts^16^,^17^,^18^,^19^, which cause severe socioeconomic damage on the adjacent continents. Our results indicate that SST seasonality in the tropical Atlantic did not substantially change during a period of abrupt high-latitude ice sheet, ocean and climate perturbations at the end of the last interglacial, and, thus, suggest that tropical SST seasonality is controlled mainly by orbital insolation changes during interglacials. However, more seasonally resolved proxy records of SST are needed to better constrain both the climate sensitivity of the tropical ocean in the past and the seasonal response in model-based scenarios of past and future climate change.

## Methods

### Screening for diagenesis

The fossil *D. strigosa* coral (BON-5-D) was screened for potential diagenetic alteration of its skeleton using X-radiography, powder X-ray diffraction, thin-section petrography and scanning electron microscope (SEM) analysis. X-radiography reveals a well-preserved skeleton, a clear pattern of alternating bands of high and low skeletal density and continuous upward growth at a rate of 0.68±0.15 cm per year (±1 s.d.) (Supplementary Fig. 1), similar to the annual density-band pairs and growth rates reported for Holocene *D. strigosa* corals from Bonaire^23^. Powder X-ray diffraction analysis indicates that the aragonitic skeleton has a calcite content of <1%. Petrographic thin sections indicate excellent preservation of primary porosity, with no evidence for significant amounts of secondary aragonite or calcite cements (Supplementary Fig. 2). SEM analysis indicates slight dissolution of more fragile skeletal elements such as septa and columella; however, the dense theca walls that are the target for our geochemical analysis^37^,^38^ are unaffected by these subtle diagenetic alterations (Supplementary Fig. 3). Overall, the fossil coral is very well preserved.

### ^230^Th/U dating

The age of the fossil *D. strigosa* coral (BON-5-D) was determined by thermal ionization mass spectrometry ^230^Th/U dating carried out at the Heidelberg Academy of Sciences, Heidelberg, Germany^39^,^40^. The ^230^Th/U age of 117.7±0.8 is reported with its 2σ error in kyr before the year of measurement, which is AD 2009 (Supplementary Table 1), a common procedure in studies of ^230^Th/U-dated last interglacial corals^7^,^11^,^12^,^21^,^22^. The age was calculated using the half-lives of ref. 41 and corrected for the effect of detrital contamination assuming a bulk earth ^232^Th/^238^U weight ratio of 3.8 and secular equilibrium between ^238^U, ^234^U and ^230^Th. However, this correction is insignificant for the Bonaire coral. The reliability of the determined ^230^Th/U age was checked using established criteria^42^, such as initial (^234^U/^238^U) in agreement with the value of modern seawater (that is, 1.1466±0.0025 (ref. 43)), ^238^U concentrations comparable to modern corals of the same species, a ^232^Th content lower than 2 p.p.b. and negligible calcite content^42^. All these criteria are fulfilled for coral BON-5-D. The coral’s ^230^Th/U-age is, thus, considered as strictly reliable. The 2σ uncertainties of the ^230^Th/U ages (Fig. 2e,f) are 0.8 kyr for the late last interglacial coral, ~0.03 kyr for the Holocene corals^23^ (ages are given relative to AD 1950) and <0.01 kyr for the modern corals^23^.

### Microsampling

*D. strigosa* coral BON-5-D was microsampled along its major growth axis by carefully drilling continuously along the centre of the dense theca walls using a 0.6-mm-diameter drill bit following established methods^38^ (Supplementary Fig. 1). The methodology is identical to our microsampling of modern and fossil Holocene *D. strigosa* corals from Bonaire^23^,^27^. An average of 11.4 samples per year was obtained, which is in the range of sampling resolutions reported for our modern and fossil Holocene *D. strigosa* corals from Bonaire (10.8–15.3 samples per year; ref. 23).

### Geochemical and isotopic analyses

Coral Sr/Ca and δ^18^O were analysed on splits of the same powder samples at MARUM (University of Bremen) as previously described^23^,^38^. Twenty-five splits of the coral reference material JCp-1 (ref. 44) were treated like samples, and the average Sr/Ca value obtained in this study was 8.919±0.008 mmol mol^−1^, which is the same JCp-1 reference composition as reported in our Holocene Bonaire coral study^23^.

### Coral record

The internal chronology of the BON-5-D coral record is based on counting the clear annual cycles in Sr/Ca and δ^18^O that reflect the SST seasonality. This age model is corroborated by the skeletal pattern of annual density-band pairs as revealed by X-radiographs. For the construction of the chronology, annual Sr/Ca maxima were set to February/March (on average the coolest months) and annual Sr/Ca minima to September/October (on average the warmest months) using the present-day SST climatology^26^ as a benchmark. The coral δ^18^O chronology uses the tie points of the coral Sr/Ca chronology. The resulting records were interpolated to monthly resolution. The methodology is similar as described for our Holocene Bonaire coral records^23^,^27^. We note that the shift in the mean coral Sr/Ca and δ^18^O that occurs between years 7 and 8 of the internal chronology (Fig. 2a,b) is not related to a change of the microsampling transect nor to any shifts in extension rate or coral δ^13^C and, consequently, likely reflects a climatic shift. Importantly, this shift does not affect the amplitude of our SST seasonality calculations described below.

### Coral seasonality

Coral Sr/Ca (δ^18^O) seasonality was calculated following established methods^10^ and similar to our Holocene Bonaire coral study^23^,^27^. Seasonality is calculated as the difference between the maximum and the minimum monthly coral Sr/Ca (δ^18^O) value of a given year. The mean seasonality is calculated by averaging the seasonality of all years of a given coral record. The uncertainty assigned to each coral-SST seasonality estimate for a given snapshot is ±1 s.e. (Fig. 2e,f). The fossil coral-SST seasonality estimates are then compared with the ±1 s.d. around the reconstructed modern mean SST seasonality based on the three modern corals. In addition, the combined error^45^ (±1 CE) is considered for each fossil coral-SST seasonality estimate (Supplementary Fig. 4), which is derived from the combination (root of the sum of the squares) of (1) the s.d. (2 s.d.) around the reconstructed modern mean SST seasonality based on the three modern corals and (2) the s.e. (2 s.e.) of the mean of multiple SST seasonality estimates for each fossil coral^23^,^27^,^45^.

### Climate model simulations

The state-of-the-art coupled atmosphere–ocean general circulation model COSMOS is applied^46^,^47^,^48^, which is also used in the 5th Assessment Report (AR5) of the Intergovernmental Panel on Climate Change. COSMOS consists of the atmosphere model ECHAM5 (ref. 49), the land–surface model JSBACH^50^, the general ocean circulation model MPIOM^51^ and the OASIS3 coupler^52^. The land-surface and vegetation model JSBACH comprise a dynamic vegetation module^53^, which enables the plant cover to adjust to a change in the climate state. The model has been tested and applied for early and mid-Holocene climates^47^,^48^, glacial climates^54^,^55^,^56^, as well as for Cenozoic climates^57^,^58^,^59^. The resolution used in our simulations is T31 (3.75°) in the atmosphere with 19 vertical levels and a horizontal resolution of 3° × 1.8° in the ocean with 40 vertical levels. The ocean grid has an effective higher resolution in the polar regions^51^,^58^. Fixed modern distributions of continental ice sheets (except for the experiment with reduced Greenland ice sheet), sea level and distribution of land were used throughout the simulations. For the transient simulations, orbital acceleration^60^ with a factor of 10 has been applied to simulate the time interval from 130 to 115 kyr ago and the last 8 kyr.

The last interglacial transient simulation starts from a quasi-equilibrated time slice run for 130 kyr ago that was spun up for 1,000 years, which has previously been analysed in a multimodel assessment of last interglacial temperatures^2^, using the greenhouse gas boundary conditions specified by the third phase of the Paleoclimate Model Intercomparison Project (PMIP3): 257 ppmv for CO_2_, 512 ppbv for CH_4_ and 239 ppbv for N_2_O. Throughout the last interglacial transient simulation, the greenhouse gas concentrations varied according to the values specified by PMIP3 (refs 61, 62, 63), which were interpolated to 0.01 kyr resolution. In accordance with PMIP3, both the spin-up and the transient simulation were performed with fixed pre-industrial vegetation.

In addition, a freshwater perturbation experiment based on the last interglacial transient simulation was performed with an identical set-up, by distributing a freshwater flux anomaly (0.02 Sv) over the North Atlantic Ocean (45° W–20° W, 40° N–55° N) that starts at 119.25 kyr ago and ends 117.76 kyr ago, representing 1,500 calendar years (150 model years). This time interval was chosen in order to have the last 500 calendar years (50 model years) of the freshwater perturbation centred at 118.00 kyr ago. The transient simulation then continues from 117.75 to 115.0 kyr ago without freshwater forcing.

Moreover, a last interglacial transient simulation with reduced Greenland ice sheet was performed. The simulation starts from a quasi-equilibrated time slice run for 130 kyr ago that was spun up for 1,500 years, which has previously been analysed in a sensitivity study on the influence of the Greenland ice sheet on last interglacial climate^64^. Throughout the spin-up and last interglacial transient simulations with reduced Greenland ice sheet, the greenhouse gas boundary conditions were fixed to pre-industrial values: 278 ppmv for CO_2_, 650 ppbv for CH_4_ and 270 ppbv for N_2_O. In the simulations, the Greenland ice-sheet elevation was reduced by subtracting 1,300 m from each grid point over Greenland. Areas where the present elevation is lower than 1,300 m were defined as ice-free and the albedo was adjusted accordingly. The spin-up and the transient simulation were performed with dynamic vegetation.

The Holocene transient simulation has previously been analysed in terms of Southern Hemisphere westerly winds evolution^65^. The Holocene transient simulation starts from a quasi-equilibrated time slice run for 8.1 kyr ago, using greenhouse gas concentrations at pre-industrial levels: 278 ppmv for CO_2_, 650 ppbv for CH_4_ and 270 ppbv for N_2_O. Throughout the Holocene transient simulation the greenhouse gas concentrations were fixed. The spin-up and the transient simulation were performed with dynamic vegetation.

## Author contributions

T.F. designed the study and wrote the manuscript; C.G. performed coral microsampling, generated coral geochemical time series and was responsible for quantification of seasonality and screening for diagenesis; D.S. was responsible for coral ^230^Th/U dating; G.L. and M.P. performed model simulations; J.P., T.F. and S.R.S. discovered the coral and drilled the core; M.K. was responsible for coral Sr/Ca analysis; T.F. was responsible for the Bonaire 2006 Expedition; S.R.S. was responsible for local logistics, local field expertise and permissions. All authors contributed to data interpretation and manuscript preparation.

## Additional information

**How to cite this article:** Felis, T. *et al.* Tropical Atlantic temperature seasonality at the end of the last interglacial. *Nat. Commun.* 6:6159 doi: 10.1038/ncomms7159 (2015).

## Supplementary Material

Supplementary Information Supplementary Figures 1-7, Supplementary Table 1, Supplementary Note 1 and Supplementary References
