# Supplementary material for: Tropical Atlantic temperature seasonality at the end of the last interglacial
Source: Nat Commun. 2015 Jan 22;6:6159. doi: 10.1038/ncomms7159 (PMC4317504; doi:10.1038/ncomms7159)
Supplement: Supplementary Information — Supplementary Figures 1-7, Supplementary Table 1, Supplementary Note 1 and Supplementary References [file ncomms7159-s1.pdf]

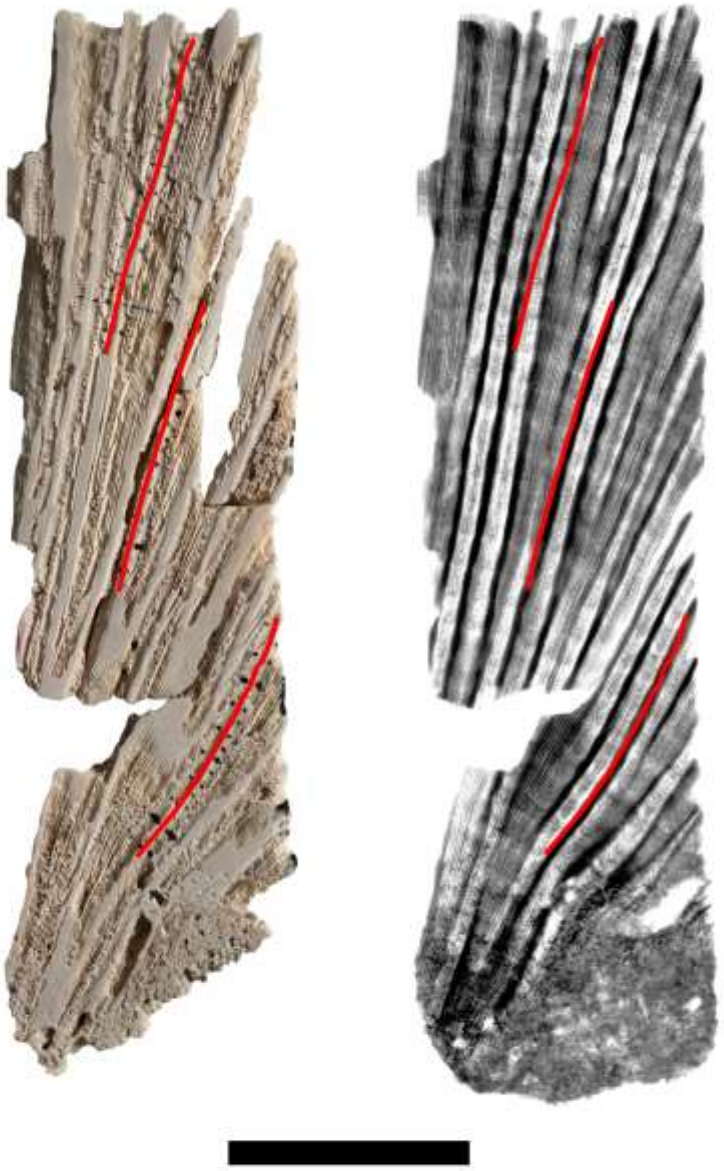

Supplementary Figure 1. Images of the fossil Bonaire coral. Photographic image (**left**) and X-radiograph positive image (**right**) of the slabbed *Diploria strigosa* coral (BON-5-D) core reveal a generally well preserved skeleton. A clear pattern of alternating horizontal bands of high (dark colour) and low skeletal density (light colour) is visible on the X-radiograph. One year is represented by a high-density/low density band pair. The microsampling transect along the dense vertical theca walls is indicated by the red line. The apparent slight visual offset of the uppermost transect from the theca results from integration of the three-dimensional skeletal structure over the depth of the 6-7 mm thick slab by the X-radiograph. Coral growth direction is toward the top of the image. The length of the scale bar is 5 cm.

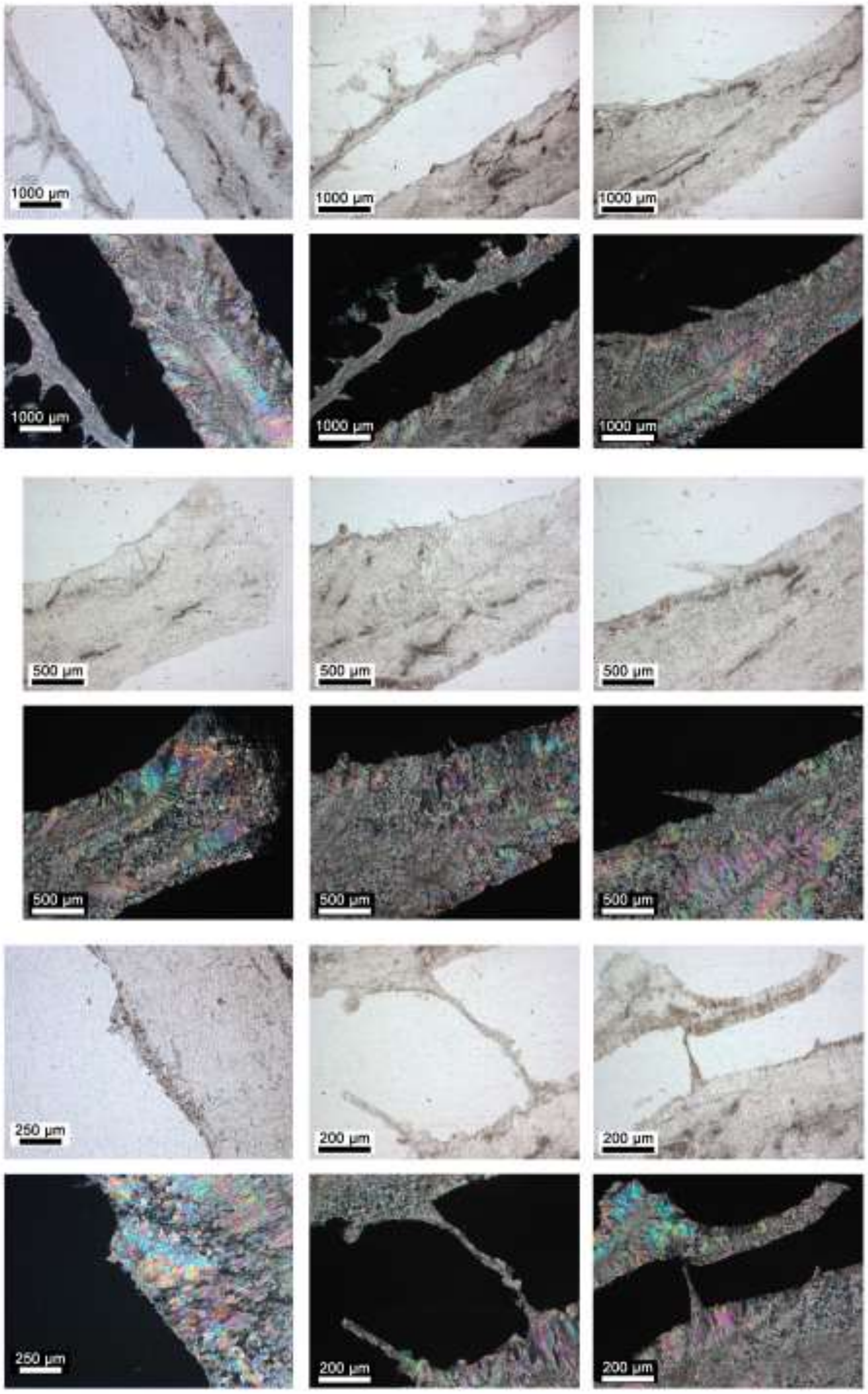

Supplementary Figure 2. Thin section photomicrographs of the fossil Bonaire coral. Thin sections of *Diploria strigosa* coral BON-5-D are shown under transmitted and cross-polarised light. No significant amounts of secondary aragonite or calcite cements are observed in the skeletal pore spaces, documenting the excellent preservation of primary porosity.

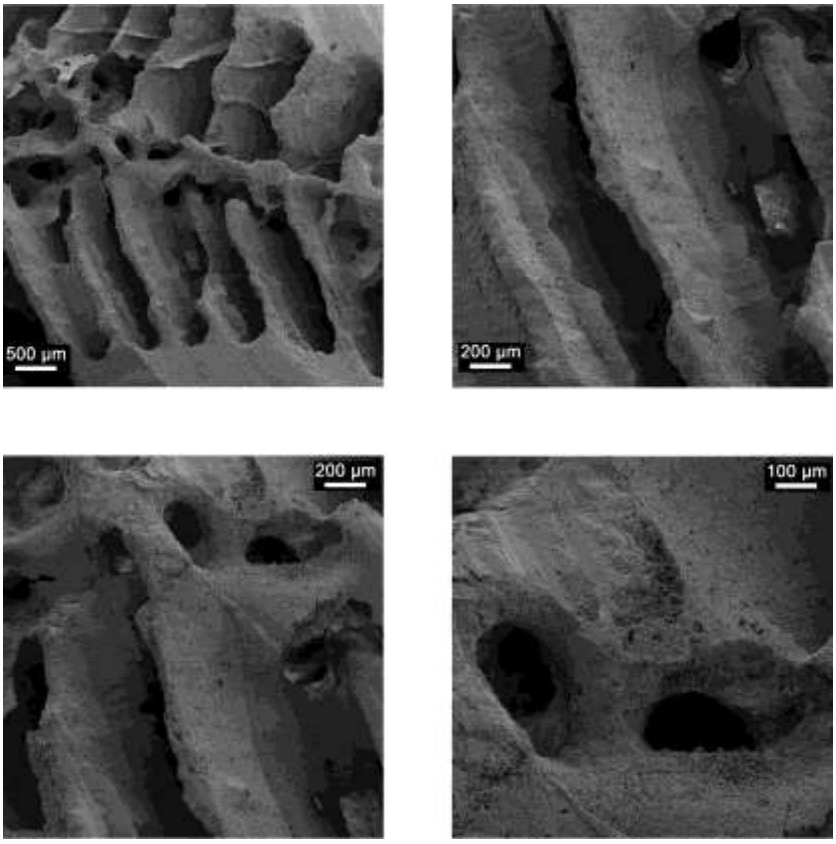

Supplementary Figure 3. Scanning electron microscope images of the fossil Bonaire coral. Scanning electron microscope (SEM) images of *Diploria strigosa* coral BON-5-D. No significant amounts of secondary aragonite or calcite cements are observed in the skeletal pore spaces, documenting the excellent preservation of primary porosity. In some areas slight dissolution of more fragile skeletal elements such as septa and columella is observed, but the dense theca walls that are the target for our geochemical analysis are unaffected by these subtle diagenetic alterations.

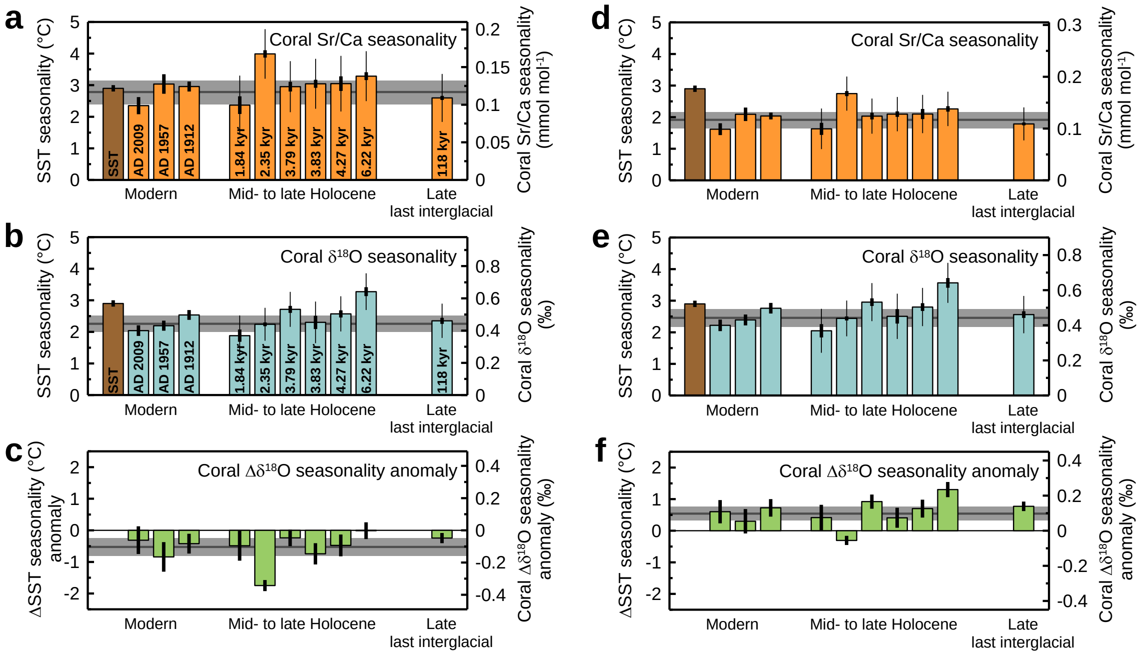

Supplementary Figure 4. Tropical North Atlantic coral-based temperature seasonality. a, Sr/Ca-based sea surface temperature (SST) seasonality from Bonaire *Diploria strigosa* corals, derived from a seasonal Sr/Ca-SST relationship (-0.042 mmol mol^{-1} per °C) for *D. strigosa*[1], and Bonaire instrumental SST seasonality (1910-2000, 2° × 2° gridbox centred at 12° N, 68° W, ERSST.v3b)[2]. The dark grey line represents the reconstructed modern mean SST seasonality based on three modern corals and the light grey bar the ±1SD around this mean. The uncertainty assigned to each SST seasonality estimate is the ±1SE (thick vertical error bar). In addition, the combined error[3] (±1CE) is shown for each fossil coral-SST seasonality estimate (thin vertical error bar), which is derived from the combination (root of the sum of the squares) of (1) the standard deviation (2SD) around the reconstructed modern mean SST seasonality based on the three modern corals, and (2) the standard error (2SE) of the mean of multiple SST seasonality estimates for each fossil coral[3-5]. **b,** The coral δ^{18}O-based SST seasonality, derived from a seasonal δ^{18}O-SST relationship (-0.196‰ per °C) for *D. strigosa*[1]. **c,** The residual coral δ^{18}O seasonality ($\Delta\delta^{18}$O) anomalies, obtained by subtracting the Sr/Ca-SST seasonality from the δ^{18}O-SST seasonality (removal of the temperature component of the δ^{18}O signal) based on the *D. strigosa* relationships[1]. The dark grey line represents the reconstructed modern mean $\Delta\delta^{18}$O seasonality anomaly based on three modern corals and the light grey bar the ±1SD around this mean. The uncertainty assigned to each $\Delta\delta^{18}$O seasonality anomaly is the $\pm(1SE_{Sr/Ca\ seasonality} + 1SE_{\delta 18O\ seasonality})$ (thick vertical error bar). Negative $\Delta\delta^{18}$O seasonality anomalies may reflect hydrologic cycle effects on seawater δ^{18}O such as winter rainfall/summer evaporation, whereas positive $\Delta\delta^{18}$O seasonality anomalies may reflect summer rainfall/winter evaporation. **d,** As in (**a**), with coral-based SST seasonality derived from a seasonal Sr/Ca-SST relationship (-0.061 mmol mol^{-1} per °C) for *Porites*[6]. **e,** As in (**b**), with coral-based SST seasonality derived from a seasonal δ^{18}O-SST relationship (-0.180‰ per °C) for *Porites*[7]. **f,** As in (**c**), with coral $\Delta\delta^{18}$O seasonality anomalies based on the *Porites* relationships[6,7]. Holocene and modern coral data are from refs 4,5.

a

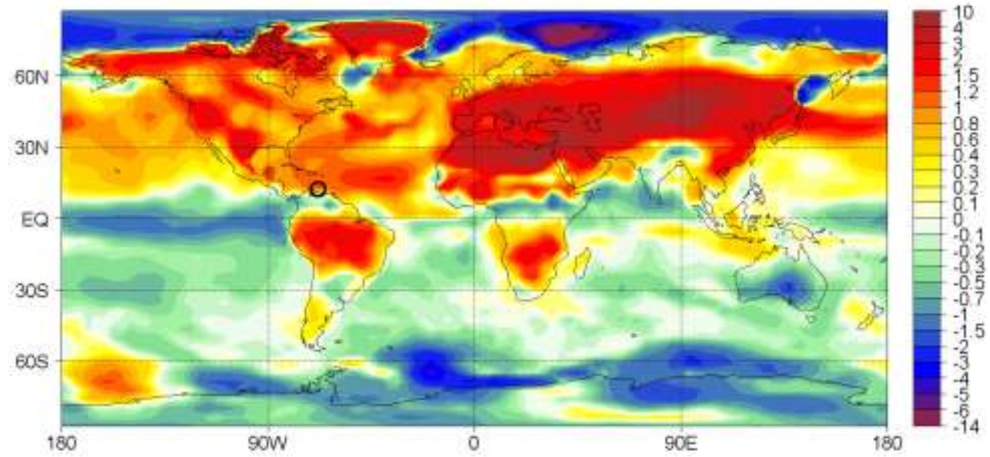
60N
30N
EQ
30S
60S
180
90W
0
90E
180
10
4
3
2
1.5
1.2
1
0.8
0.6
0.4
0.3
0.2
0.1
0
-0.1
-0.2
-0.3
-0.5
-0.7
-1
-1.5
-2
-3
-4
-5
-6
-14

b

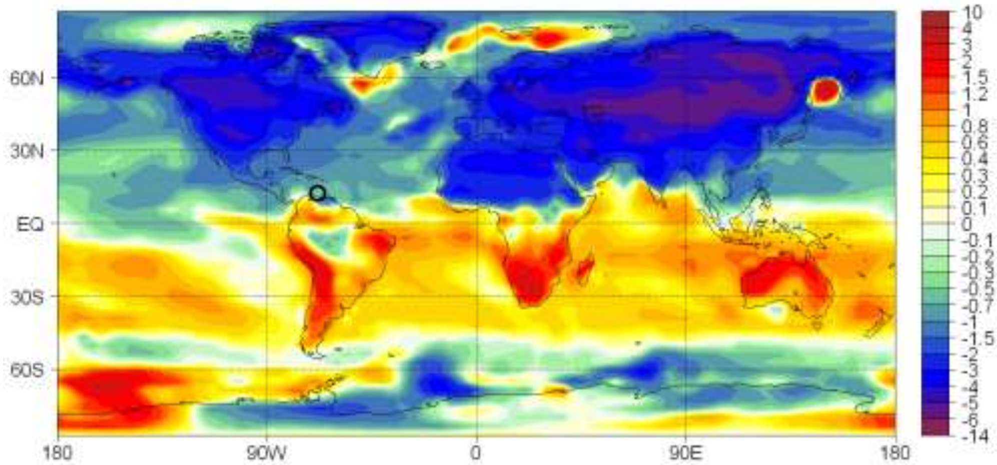
60N
30N
EQ
30S
60S
180
90W
0
90E
180
10
4
3
2
1.5
1.2
1
0.8
0.6
0.4
0.3
0.2
0.1
0
-0.1
-0.2
-0.3
-0.5
-0.7
-1
-1.5
-2
-3
-4
-5
-6
-14

c

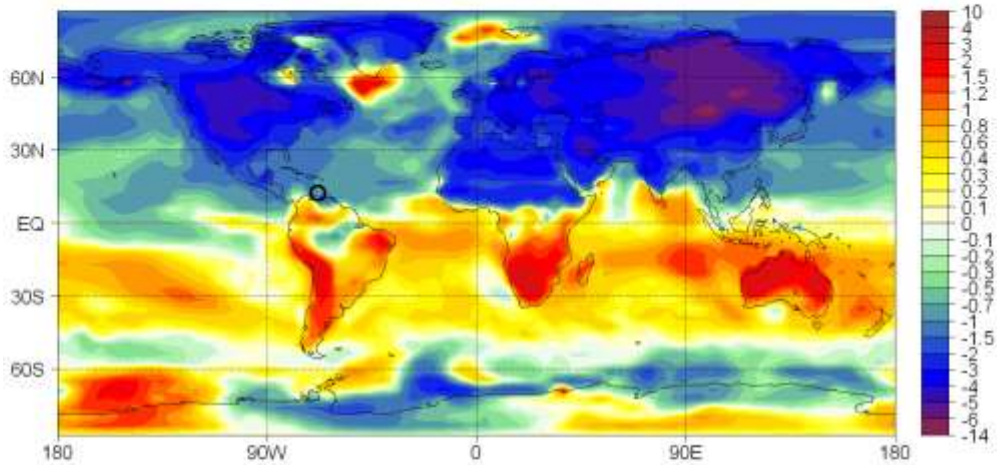
60N
30N
EQ
30S
60S
180
90W
0
90E
180
10
4
3
2
1.5
1.2
1
0.8
0.6
0.4
0.3
0.2
0.1
0
-0.1
-0.2
-0.3
-0.5
-0.7
-1
-1.5
-2
-3
-4
-5
-6
-14

d

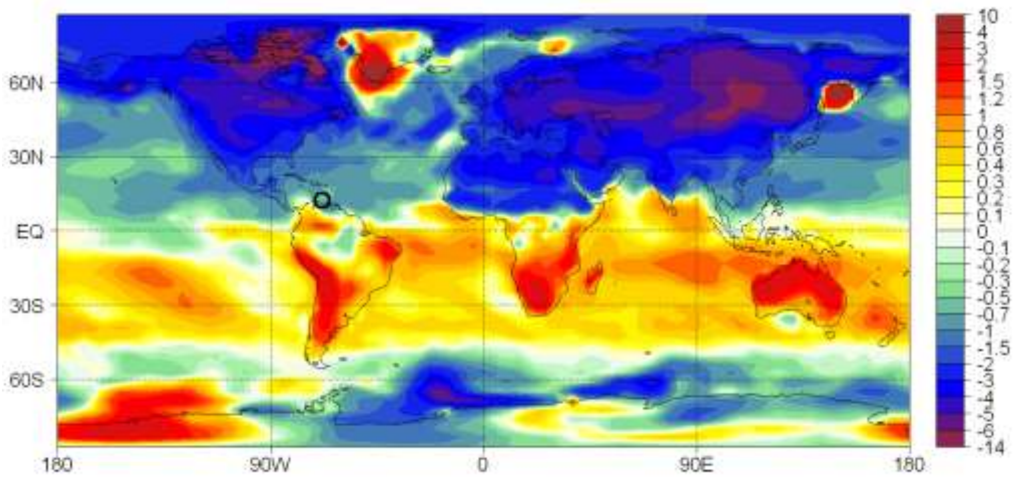

Supplementary Figure 5. Model-based surface air temperature seasonality anomalies for 6 kyr and 118 kyr ago. The simulations are based on the coupled atmosphere-ocean general circulation model COSMOS. Seasonality is derived as the difference of the warmest minus the coolest temperature at a given gridbox. **a**, The difference in near-surface air temperature seasonality between mid-Holocene (6 kyr ago) and pre-industrial climate is shown. **b,** The difference in near-surface air temperature seasonality between late last interglacial (118 kyr ago) and pre-industrial climate is shown. **c,** The difference in near-surface air temperature seasonality between late last interglacial (118 kyr ago) and pre-industrial climate is shown for the freshwater hosing experiment. **d,** The difference in near-surface air temperature seasonality between late last interglacial (118 kyr ago) and pre-industrial climate is shown for the reduced Greenland ice sheet experiment. Each time slice represents the mean of 50 model years. Units are °C. The location of our coral site at Bonaire is indicated (black circle).

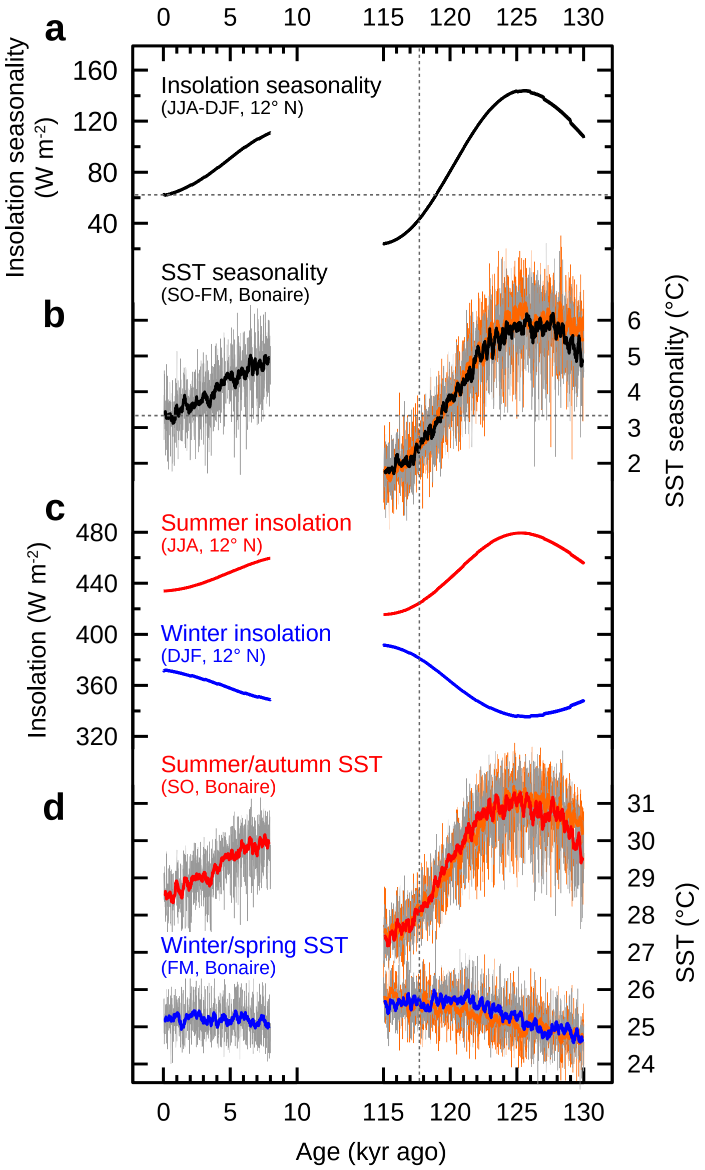

Supplementary Figure 6. Tropical North Atlantic insolation and temperature changes. a, Insolation seasonality[8] at the latitude of Bonaire, calculated as difference of boreal summer (June-July-August, JJA) minus winter insolation (December-January-February, DJF). **b,** Sea surface temperature (SST) seasonality at Bonaire simulated by the coupled atmosphere-ocean general circulation model COSMOS (1° × 1° gridbox centred at 12.5° N, 68° W), derived from the difference of simulated summer/autumn (September-October, SO) minus winter/spring (February-March, FM) SST. **c,** Summer (JJA) and winter (DJF) insolation[8] at the latitude of Bonaire. **d,** Summer/autumn (SO) and winter/spring (FM) SST at Bonaire simulated by COSMOS. Bold line (**b,d**) represents a 21-point running average, representing an average of 210 calendar years. Results of the reduced Greenland ice sheet experiment are also shown (orange). Dashed horizontal lines (**a,b**) indicate the modern value for insolation and simulated SST seasonality. Dashed vertical line indicates the Bonaire coral age (117.7 ± 0.8 kyr).

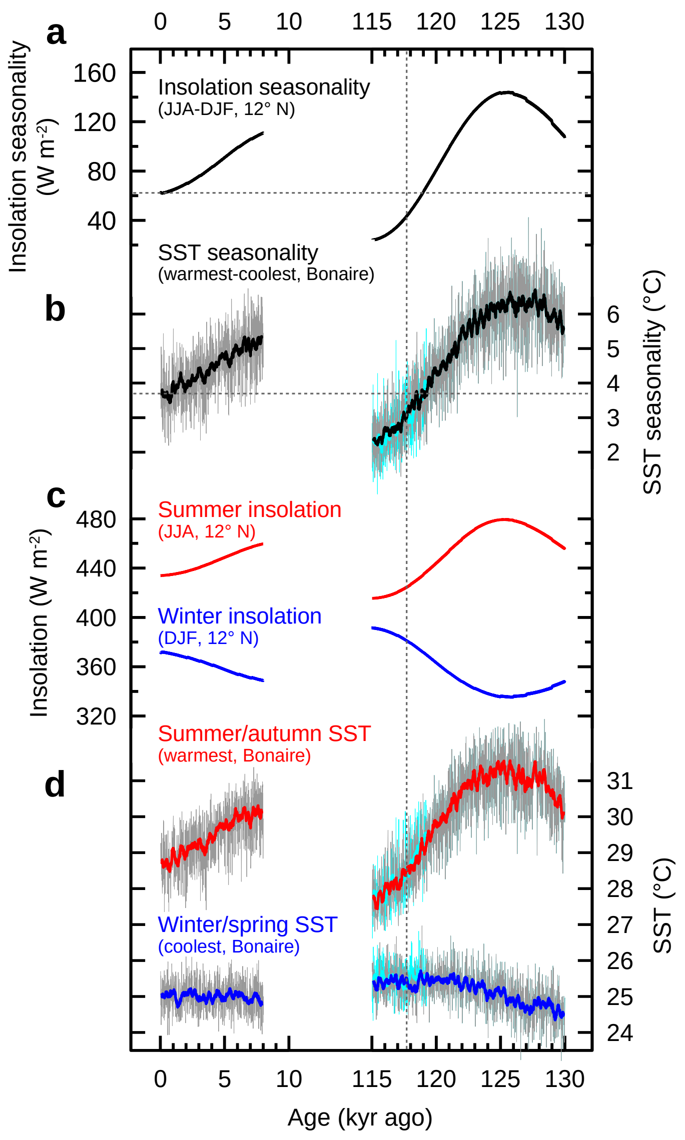

Supplementary Figure 7. Tropical North Atlantic insolation and temperature changes. a, Insolation seasonality[8] at the latitude of Bonaire, calculated as the difference of boreal summer (June-July-August, JJA) minus winter insolation (December-January-February, DJF). **b,** Sea surface temperature (SST) seasonality at Bonaire simulated by the coupled atmosphere-ocean general circulation model COSMOS (1° × 1° gridbox centred at 12.5° N, 68° W), derived from the difference of simulated summer/autumn (warmest) minus winter/spring (coolest) SST. The SST seasonality evolution is very similar to that derived from the difference of September-October minus February-March SST (Fig. 3). **c,** Summer (JJA) and winter (DJF) insolation[8] at the latitude of Bonaire. **d,** Summer/autumn (warmest) and winter/spring (coolest) SST at Bonaire simulated by COSMOS. Bold line (**b,d**) represents a 21-point running average, representing an average of 210 calendar years. Results of the freshwater hosing experiment are also shown (light blue). Dashed horizontal lines (**a,b**) indicate the modern value for insolation and simulated SST seasonality. Dashed vertical line indicates the Bonaire coral age (117.7 ± 0.8 kyr).

Supplementary Table 1. Results of ^{230}Th/U-dating of the Bonaire fossil coral.

Sample	^{238}U [µg/g]	±	^{232}Th [ng/g]	±	(^{234}U/^{238}U)	±	(^{230}Th/^{238}U)	±	$Age_{uncorrected}$ [kyr]	±	$Age_{corrected}$ [kyr]	±	(^{234}U/^{238}U)$_{initial}$	±
**BON-5-D**	2.863	0.003	1.957	0.004	1.1071	0.0016	0.7411	0.0028	117.7	0.8	117.7	0.8	149.4	2.1

The age is given in thousand years (kyr) before the year of measurement (AD 2009). All reported errors are given at the 2σ level.

Supplementary Note 1

Coral Sr/Ca as sea surface temperature seasonality proxy

Coral skeletal Sr/Ca is a powerful proxy for reconstructing past changes in sea surface temperature (SST) variability[6,7,9]. Studies using at least 3 modern corals for a given reef site, to assess between-colony differences, show that sub-seasonally resolved Sr/Ca records satisfactorily document the instrumental SST seasonality of the subtropical to tropical ocean[4,10,11]. The studies find a reconstructed modern Sr/Ca-SST seasonality that ranges from 4.5 to 5.6 °C (5.4 °C instrumental SST seasonality) at the northern Gulf of Aqaba[10], from 2.2 to 3.2 °C (2.6 °C instrumental SST seasonality) at Tahiti[11], and from 2.4 to 3.0 °C (2.9 °C instrumental SST seasonality) at Bonaire[4]. At a given site, these between-colony differences may be partly due to real differences in SST seasonality representing different shallow-water habitats on the reef, the sampling of different precise time intervals, and, partly due to between-colony differences in skeletal mass accumulation during skeletogenesis within the living tissue layer[12]. Crucially, the Gulf of Aqaba study[10] finds an increased Sr/Ca-SST seasonality of 8.4 °C recorded in a fossil 122-kyr-old last interglacial coral, compared with the reconstructed modern mean SST seasonality of 5.2 °C at this site, demonstrating the potential of coral Sr/Ca as SST seasonality proxy on orbital timescales.

Supplementary References

1. Hetzinger, S., Pfeiffer, M., Dullo, W.-C., Ruprecht, E. & Garbe-Schönberg, D. Sr/Ca and $\delta^{18}O$ in a fast-growing *Diploria strigosa* coral: Evaluation of a new climate archive for the tropical Atlantic. *Geochem. Geophys. Geosyst.* **7**, Q10002 (2006).
2. Smith, T. M., Reynolds, R. W., Peterson, T. C. & Lawrimore, J. Improvements to NOAA's historical merged land-ocean surface temperature analysis (1880-2006). *J. Clim.* **21**, 2283-2296 (2008).
3. Abram, N. J., McGregor, H. V., Gagan, M. K., Hantoro, W. S. & Suwargadi, B. W. Oscillations in the southern extent of the Indo-Pacific Warm Pool during the mid-Holocene. *Quat. Sci. Rev.* **28**, 2794-2803 (2009).

4. Giry, C. *et al.* Mid- to late Holocene changes in tropical Atlantic temperature seasonality and interannual to multidecadal variability documented in southern Caribbean corals. *Earth Planet. Sci. Lett.* **331–332**, 187-200 (2012).
5. Giry, C. *et al.* Controls of Caribbean surface hydrology during the mid- to late Holocene: insights from monthly resolved coral records. *Clim. Past* **9**, 841-858 (2013).
6. Corrège, T. Sea surface temperature and salinity reconstruction from coral geochemical tracers. *Palaeogeogr., Palaeoclimatol., Palaeoecol.* **232**, 408-428 (2006).
7. Gagan, M. K. *et al.* Temperature and surface-ocean water balance of the mid-Holocene tropical western Pacific. *Science* **279**, 1014-1018 (1998).
8. Berger, A. L. Long-term variations of daily insolation and Quaternary climatic changes. *J. Atmos. Sci.* **35**, 2362-2367 (1978).
9. Beck, J. W. *et al.* Sea-surface temperature from coral skeletal strontium/calcium ratios. *Science* **257**, 644-647 (1992).
10. Felis, T. *et al.* Increased seasonality in Middle East temperatures during the last interglacial period. *Nature* **429**, 164-168 (2004).
11. Felis, T. *et al.* Pronounced interannual variability in tropical South Pacific temperatures during Heinrich Stadial 1. *Nat. Commun.* **3**, 965, doi:10.1038/ncomms1973 (2012).
12. Gagan, M. K., Dunbar, G. B. & Suzuki, A. The effect of skeletal mass accumulation in *Porites* on coral Sr/Ca and $\delta^{18}O$ paleothermometry. *Paleoceanography* **27**, PA1203 (2012).
